# Supplementary material for: Characterization of LysBC17, a Lytic Endopeptidase from Bacillus cereus
Source: Antibiotics (Basel). 2019 Sep 19;8(3):155. doi: 10.3390/antibiotics8030155 (PMC6784087; doi:10.3390/antibiotics8030155)
Supplement: Supplementary file 1 [file antibiotics-08-00155-s001.pdf]

Supplemental Information

Characterization of LysBC17, a lytic endopeptidase from *Bacillus cereus*

Steven M. Swift, Irina V. Etobayeva, Kevin P. Reid, Jerel J. Waters, Brian B. Oakley,

David M. Donovan, and Daniel C. Nelson

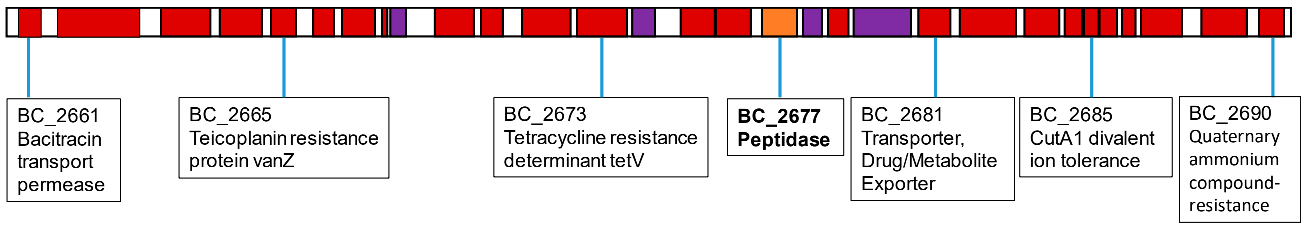

Supplemental Figure 1. Drug and metal resistance genes near the BC_2677 locus in the *B. cereus* ATCC 14579 genome (GenBank: AE016877.1). The 32 kb window displayed covers nucleotides 2643000 – 2675000. No holin gene or other phage genes are nearby. The BC_2677 gene does not encode a signal peptide. This suggests BC_2677 (LysBC17) is an autolysin. Orange and red boxes are coding regions in the same orientation, and purple boxes are coded on the complementary DNA strand.

```
PlyBC17   1 MKYHNRNVSNLNKLADNTKAAAFKWYQYCIDNGIEVLIYETIRTVEQQREYVRKGASQTM
Wip1      1 MYYHNRNLANLEKLAPHTRQKAKQWYQYCVENGIEVLIYETTRTIEQQRENVRKGASQTM


PlyBC17  61 RSYHLVGQALDFVPIQSNGTEDWNGYNKEPWASAIRYAKQIGFEWGGDWKGFVDSPHLQY  120
Wip1     61 KSYHLVGQALDFVPARE-AEVYWDGYYRNDIQKAIGYAKSIGFEWGGDWKGFVDSPXLQY  119
```

Supplemental Figure 3. Clustal Omega alignment and BoxShade view of the LysBC17 catalytic domain (residues 1-120) and the *Bacillus* phage Wip1 endolysin catalytic domain (YP_008433324, residues 1-119). Black boxes represent identity and grey boxes represent similarity.

```
PlyBC17      131 GKGA-QNVVTPPTSNDNVGVAYINGSNVNLRKGPGTGYGVIRQLGKGESYKVFGQTNSWL
Waukesha92   160 GGGSPVMPTPIPPSNDGTKVAYINGDNVNLRKGPGTGYAVIRKLGKGECYQVWGESNGWL


PlyBC17      190 NLGGDQWVYNDPSYIRYTGGNVPATSQSSNDGVGVVTIIADVLRVRTGPGTNYGIVKNVY
Waukesha92   220 NLGGDQWVYNDSSYIRYTGENAPAPSKPSNDGIGVVTITADVLRVRTGPGTNYGVVKNVY


PlyBC17      250 QGEKYQSFGYKDGWYNVGGNQWVSGEYVTFVK  281
Waukesha92   280 QSERYQSWGYRDGWYNVGGDQWVSGEYVKFEK  311
```

Supplemental Figure 2. Clustal Omega alignment and BoxShade view of the LysBC17 binding domain (residues 231-281) and the *Bacillus* phage Waukesha92 endolysin binding domain (YP_009099314, residues 160-311). Black boxes represent identity and grey boxes represent similarity.

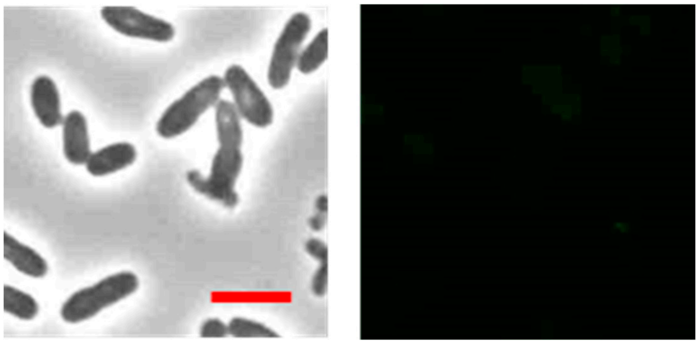

Supplemental Figure 4. LysBC17 GFP-CWB binding to *B. cereus* ATCC 13061. Bacterial cells and GFP-labeled LysBC17 CWB were mixed together, washed, and viewed by microscopy per the Methods. 1000X phase-contrast image (left panel) is shown with its corresponding fluorescent images (right panel). Exposure time for the fluorescent image was 100 ms, identical to the time used in Figure 5. Scale bar = 5 μm.
